# An insight into the role of phosphotransacetylase (*pta*) and the acetate/acetyl-CoA node in *Escherichia coli*

**DOI:** 10.1186/1475-2859-8-54

**Published:** 2009-10-24

**Authors:** Sara Castaño-Cerezo, José M Pastor, Sergio Renilla, Vicente Bernal, José L Iborra, Manuel Cánovas

**Affiliations:** 1 Department of Biochemistry and Molecular Biology B and Immunology, Campus de Espinardo, Universidad de Murcia, E-30100, Spain

## Abstract

**Background:**

Acetate metabolism in *Escherichia coli *plays an important role in the control of the central metabolism and in bioprocess performance. The main problems related to the use of *E. coli *as cellular factory are i) the deficient utilization of carbon source due to the excretion of acetate during aerobic growth, ii) the inhibition of cellular growth and protein production by acetate and iii) the need for cofactor recycling (namely redox coenzymes and free CoASH) to sustain balanced growth and cellular homeostasis.

**Results:**

This work analyzes the effect of mutations in the acetate excretion/assimilation pathways, acetyl-CoA synthethase (*acs*) and phosphotransacetylase (*pta*), in *E. coli *BW25113 grown on glucose or acetate minimal media. Biomass and metabolite production, redox (NADH/NAD^+^) and energy (ATP) state, enzyme activities and gene expression profiles related to the central metabolism were analyzed. The *knock-out *of *pta *led to a more altered phenotype than that of *acs*. Deletion of *pta *reduced the ability to grow on acetate as carbon source and strongly affected the expression of several genes related to central metabolic pathways.

**Conclusion:**

Results showed that *pta *limits biomass yield in aerobic glucose cultures, due to acetate production (overflow metabolism) and its inefficient use during glucose starvation. Deletion of *pta *severely impaired growth on acetate minimal medium and under anaerobiosis due to decreased acetyl-coenzyme A synthethase, glyoxylate shunt and gluconeogenic activities, leading to lower growth rate. When acetate is used as carbon source, the joint expression of *pta *and *acs *is crucial for growth and substrate assimilation, while *pta *deletion severely impaired anaerobic growth. Finally, at an adaptive level, *pta *deficiency makes the strain more sensitive to environmental changes and de-regulates the central metabolism.

## Background

When *E. coli *grows on excess of glucose it excretes acetate, a phenomenon known as the Crabtree Effect or "acetate overflow", and which has several causes. When glucose is in excess, the TCA cycle is limited, acetyl-CoA accumulates and 15-30% is excreted as acetate, allowing the regeneration of CoASH [1-3], although it is not clear how the regulation works [4]

**Figure 1:**
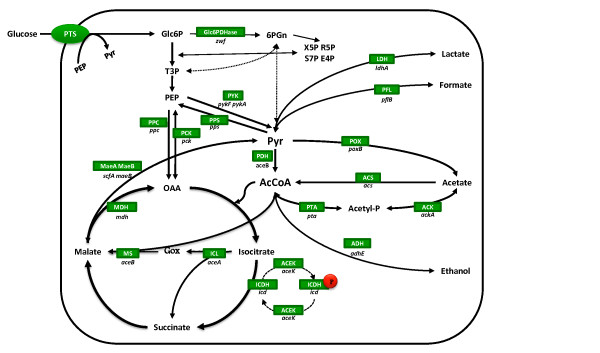
**Simplified model for the central metabolic network of E. coli metabolism**. The enzymes involved (and their codifying genes) are shown in the figure, ACEK (*aceK*), isocitrate dehydrogenase phosphatase/kinase; ACK (*ackA*), acetate kinase; ACS (*acs*), acetyl-CoA synthetase; ICDH (*icd*), isocitrate dehydrogenase; ICL (*aceA*), isocitrate lyase; ICLR (*iclR*), repressor of the glyoxylate shunt; MDH (*maeB*), malate dehydrogenase; ME (*sfcA*), malic enzyme, PFL (*pfl*) pyruvate:formate lyase; PTA (*pta*), phosphotransacetylase (Ecocyc-Metacyc [45]).

Moreover, *E. coli *is also able to metabolize the acetate produced and even use it as sole carbon source. The enzymes intervening (Figure 1) are acetyl-CoA synthetase (Acs, non-reversible dissimilation) and phosphotransacetylase-acetate kinase (Pta-Ack, reversible dissimilation). It has previously been demonstrated that Pta-AckA and Acs are the sole pathways responsible for acetate assimilation, since a double *pta/acs *deletion mutant does not grow on acetate medium [5]. Acs irreversibly transforms acetate to acetyl-CoA, consuming ATP [6]. This is a high affinity pathway with a low K_m _for acetate (200 μM) and low V_m_, and therefore suitable for metabolizing low concentrations of acetate [5]. When growing on glucose, it is expressed during the stationary phase, although its regulation is complex, since it involves several transcription factors, two sigma factors and two promoters [7]. The gene coding this enzyme, *acs*, is within an operon, together with another two genes: *yjcH*, an inner membrane conserved protein of unknown function, and *actP*, which codes for an acetate permease [8,9]. On the other hand, the phosphotransacetylase-acetate kinase (Pta-Ack) pathway is characterized by its reversibility. Under aerobiosis, this pathway excretes acetate and produces ATP, a mechanism known as "acetate overflow" [10]. It is characterized by its low affinity (K_m _7-10 mM), but high V_m _[11], thus being able to dissimilate high concentrations of acetate. These two genes belong to the same operon [12] and are constitutively expressed, although *ackA *is slightly activated by Fnr [13]. The reaction catalyzed by these two enzymes generates an unstable intermediate, acetyl-phosphate (Figure 1), which phosphorylates proteins related to signal transduction pathways [14], such as the double component systems. *In vivo*, acetyl-phosphate acts on CheY-CheA, related to the flagellar function [15] and PhoB-PhoR, which are phosphate concentration regulators [16]. Additionally, mutations on *pta *and/or *ackA *have been demonstrated to affect repair-deficient mutants of *E. coli *[17].

Besides acting as substrate or product in a large number of reactions, acetyl-CoA connects the glycolysis and the acetate metabolism pathways (namely, Acs and Pta-Ack) with the TCA cycle and the glyoxylate shunt. Thus, this metabolite is a key factor in determining biomass synthesis, the redox balance and energy yield. Moreover, the acetate/acetyl-CoA node also largely determines the control exerted by the central metabolism on the performance of many microbial-based bioprocesses. Many authors have reported the decreased efficiency of acetate over-producing strains for the high-yield production of recombinant proteins [18] and also acetate affects biotransformations [19]. In addition, acetyl-CoA is the precursor of many biosynthetic pathways [20], and the engineering of the acetyl-CoA/CoA ratio (cofactor engineering) has been demonstrated to be a valuable strategy for metabolic engineering [21,22]. In fact, the main problems related to the use of *E. coli *as cellular factory are i) the loss of carbon in the form of acetate during aerobic growth at high growth rates, ii) the inhibition of cellular growth and protein production by acetate [2] and iii) the need for cofactor recycling, (namely redox coenzymes and free CoASH) to sustain balanced growth and cellular homeostasis. Moreover, the role of acetate production and CoASH regeneration in the central metabolism of *E. coli *remains to be unveiled.

In this work, we provide a further insight into the connection between the acetate/acetyl-CoA node and the central metabolism of *E. coli*. The behaviour of *E. coli *BW25113 strains carrying deletions in the acetate assimilation/excretion pathways (Acs and Pta-Ack) was studied based on the analysis of growth and metabolite production/consumption, the energy and redox state and the expression of genes and enzyme activities related to the central metabolism. The strains were analyzed in three different metabolic scenarios.

## Results

The effect of *pta *and *acs *deletion was assessed in three different scenarios: (i) aerobic growth on glucose, (ii) aerobic growth on acetate and (iii) anaerobic growth on glucose. The parent strain, *E. coli *BW25113, was used as control (Table 1).

**Table 1:** Strains used in this work.

**Strain**	**Reference**	**Genotype**	**Antibiotics**
*E. coli *BW25113	[61]	*rrnB3 *Δ*lacZ4787 hsdR514*Δ(*araBAD*)*567 *Δ(*rhaBAD)568 rph-1*	None
*E. coli *BW25113 Δ*acs*	[47]	[BW25113] Δ*acs*	Kanamycin
*E. coli *BW25113 Δ*pta*	[47]	[BW25113] Δ*pta*	Kanamycin

### Glucose as the carbon source in aerobic batch cultures

#### Kinetics of cell growth

**Figure 2:**
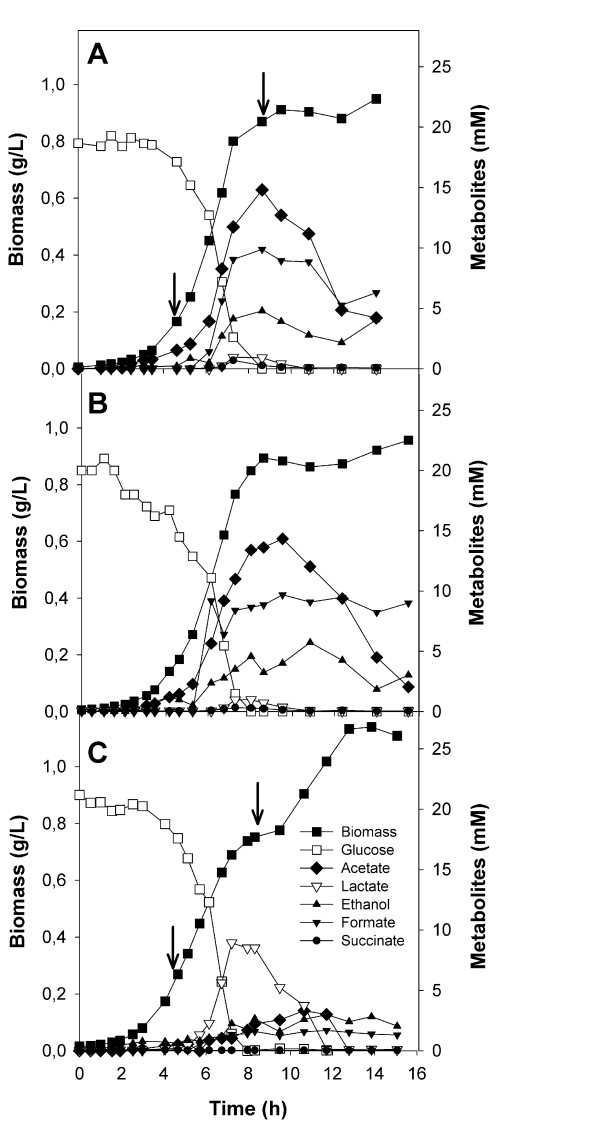
**Growth and metabolite production of A) *E. coli *BW25113 (wild type.) and its B) Δ*acs *and C) Δ*pta *knockout derivative strains**. Experiments were performed using glucose as the carbon source. Sampling times for enzyme activities and/or gene expression are indicated by arrows. Assays were carried out as indicated in the Materials and Methods section.

The growth and metabolism of *E. coli *BW25113 and its knockout strains, Δ*pta *and Δ*acs*, were characterized in aerobic cultures on glucose minimal medium. None of the mutations impaired growth; similar growth rates were observed for all the three strains, while biomass yield was slightly higher for the Δ*pta *mutant (Table 2). The production of lactate during the exponential growth phase in this mutant was nearly 10-fold higher than that of the control. Glucose exhaustion caused a lag in growth and lactate began to be consumed, leading to diauxic growth (Figure 2). The Δ*pta *mutant presented lower metabolite production rates than the *E. coli *wild type and Δ*acs *strains, and acetate production was 14-fold lower in this strain. However, residual acetate production indicated that pathways other than the Pta-AckA were active. Acetate was always consumed by all three strains in the stationary phase, but only after glucose/lactate exhaustion (Figure 2). Substantial differences were observed in the Δ*pta *mutant and further analyses were performed.

**Table 2:** Metabolic and growth parameters during growth on glucose minimal medium and aerobiosis.

**Growth parameters**	**BW25113**	**Δ*pta***	***Δacs***
**Biomass yield (g·g^-1^)**	0.27 ± 0.06	0.31 ± 0.02	0.26 ± 0.06
**Growth rate (h^-1^)**	0.74 ± 0.03	0.76 ± 0.05	0.74 ± 0.03
**Glucose uptake rate [mmol·(g·h)^-1^]**	10.64 ± 0.96	13.60 ± 0.91	11.31 ± 1.21
**Acetate production rate [mmol·(g·h)^-1^]**	7.70 ± 0.62	0.52 ± 0.07	8.60 ± 0.49
**Ethanol production rate [mmol·(g·h)^-1^]**	2.63 ± 0.27	1.23 ± 0.28	2.36 ± 0.01
**Formate production rate [mmol·(g·h)^-1^]**	7.20 ± 0.58	1.18 ± 0.01	7.78 ± 0.01
**Lactate production rate [mmol·(g·h)^-1^]**	0.71 ± 0.02	7.31 ± 0.54	0.74 ± 0.16

Culture conditions are expressed in the Materials and Methods section.

#### Energetic and redox state

The energetic and redox states of the wild type and Δ*pta *strains were determined during the early exponential phase of growth and at the onset of the stationary phase. The intracellular ATP concentration of the Δ*pta *strain was higher than that of the wild type strain, especially in the late exponential phase (Table 3). On the other hand, the redox state of both strains was nearly the same in the exponential phase and slightly reduced in the Δ*pta *strain at the onset of the stationary phase (Table 3).

**Table 3:** Energetic and redox state in *E. coli *BW25113 and its *Δpta *knockout strain.

	**ATP**		**NADH/NAD^+^**	
	**BW25113**	**Δ*pta***	**BW25113**	**Δ*pta***
	
**Glucose aerobic** **Early exponential phase**	2.27 ± 0.17	2.68 ± 0.11	0.184 ± 0.001	0.173 ± 0.003

**Glucose aerobic** **Stationary phase**	0.77 ± 0.05	1.22 ± 0.12	0.224 ± 0.003	0.262 ± 0.016

**Acetate aerobic** **Early exponential phase**	2.22 ± 0.53	2.30 ± 0.58	0.240 ± 0.007	0.260 ± 0.001

**Glucose anaerobic** **Early exponential phase**	1.04 ± 0.11	1.85 ± 0.04	0.303 ± 0.044	0.450 ± 0.084

Culture conditions are expressed in the Materials and Methods section.

#### Enzyme activities

Nine enzyme activities related to the central metabolism were studied in the early exponential phase and at the onset of the stationary phase of growth (Table 4). Pyruvate kinase (Pyk) and pyruvate dehydrogenase (Pdh) were up-regulated with the entry into the stationary phase, both activities being higher in the Δ*pta *strain in both stages. As regards the TCA cycle, Icdh was up-regulated in the Δ*pta *mutant while the first enzyme of the glyoxylate shunt (Icl) was down-regulated in the deleted strain in both phases. Taken together, these changes clearly reflect a more active glycolysis and TCA cycle in the mutant strain as a consequence of the mutation and a down-regulation of the glyoxylate shunt, as further supported by the higher Icdh/Icl ratios.

**Table 4:** Enzyme activities in the control and Δ*pta *mutant strain.

	**Glucose aerobic** **batch cultures**				**Acetate aerobic** **batch cultures**				**Glucose anaerobic** **batch cultures**	
	***Exponential phase***		***Stationary phase***		***Exponential phase***		***Stationary phase***		***Exponential phase***	

	**BW25113**	**pta^-^**	**BW25113**	**pta^-^**	**BW25113**	**pta^-^**	**BW25113**	**pta^-^**	**BW25113**	**pta^-^**

**MDH**	132.6 ± 3.1	124.0 ± 25.2	66.7 ± 17.7	74.3 ± 0.4	41.7+10.0	53.9 ± 6.8	57.3 ± 2.2	62.2 ± 5.6	71.6 ± 3.6	60.3 ± 8.7
**ICDH**	298.3 ± 3.4	432.6 ± 16.7	351.5 ± 51.0	502.2 ± 21.6	376.2 ± 14.4	243.7 ± 33.0	318.5 ± 8.6	158.4 ± 5.5	129.3 ± 4.0	136.6 ± 18.8
**ICL**	18.4 ± 0.8	15.1 ± 2.3	15.2 ± 3.7	9.6 ± 0.8	276.2 ± 31.9	187.2 ± 4.4	242.7 ± 27.4	211.4 ± 23.7	12.1 ± 0.5	5.9 ± 0.9
**ACS**	n.a.	n.a.	18.0 ± 1.4	5.6 ± 0.1	28.4 ± 9.8	0.3 ± 0.5	10.5 ± 2.3	4.6 ± 0.2	101.6 ± 1.9	10.0 ± 0.1
**PTA**	897.7 ± 10.3	n.a.	1716.7 ± 168	n.a.	1140.0 ± 39.1	n.a.	2441.8 ± 121.8	n.a.	1691.8 ± 231.2	n.a.
**POX**	240.6 ± 1.65	164.6 ± 16.6	231.5 ± 45.9	261.0 ± 14.1	323.6 ± 34.5	431.3 ± 21.7	441.05 ± 24.3	481.4 ± 43.8	251.9 ± 25.1	205.1 ± 8.2
**PDH**	249.3 ± 3.9	505.9 ± 61.8	293.6 ± 39.4	605.8 ± 106.3	141.3 ± 39.6	96.7 ± 15.4	204.5 ± 5.6	126.3 ± 25.8	211.1 ± 29.0	273.0 ± 16.2
**PYK**	184.6 ± 51.9	303.4 ± 59.9	273.9 ± 65.3	377.5 ± 17.4	189.6 ± 21.9	176.8 ± 15.9	257.2 ± 8.7	315.0 ± 14.2	461.9 ± 61.9	512.1 ± 17.6
**ZWF**	124.1 ± 23.1	140.1 ± 7.8	117.4 ± 33.0	173.9 ± 24.3	68.5 ± 14.3	100.2 ± 23.1	82.0 ± 22.5	128.6 ± 4.4	232.2 ± 30.6	129.0 ± 10.7
**ICDH/ICL**	16.2 ± 0.8	28.7 ± 4.5	23.1 ± 3.6	52.6 ± 4.8	1.4 ± 0.2	1.3 ± 0.2	1.3 ± 0.2	0.7 ± 0.1	10.7 ± 0.5	23.2 ± 4.7
**PTA/ACS**	n.a.	n.a.	95.4 ± 4.5	n.a.	40.1 ± 13.9	n.a.	231.5 ± 51.2	n.a.	16.6 ± 2.3	n.a.

Samples were taken at mid-exponential phase and at the beginning of the stationary phase. All enzyme activities are expressed as mU·mg^-1 ^protein. Experimental conditions are expressed in the Materials and Methods section. (*n.a.- *not applicable; *n.d.- *not detected).

When considering the acetate metabolism, Acs was only detected in the stationary phase of both strains, although expression in the Δ*pta *mutant was three-fold lower, probably as a result of low acetate levels (Table 4). Moreover, although the only active acetate-producing enzyme (PoxB) was expressed at high levels in both strains, low acetate production levels were observed in the Δ*pta *strain (Figure 2).

#### Gene expression by qRT-PCR

The relative expression of 29 genes related to the central metabolism of *E. coli *was analyzed at the early exponential phase and at the onset of the stationary phase in the wild type and Δ*pta *strains. The results are shown in Table 5.

**Table 5:** Relative gene expression in *E. coli *BW25113 Δ*pta *strain.

			**Glucose aerobic** **batch cultures**		**Acetate aerobic** **batch cultures**	
	***gene***	***pathway***	***Early exponential*** ***phase***	***Late exponential*** ***phase***	***Early exponential*** ***phase***	***Late exponential*** ***phase***

***rpoD***	RNA polymerase sigma 70 subunit	Transcriptional regulators	0.05 ± 0.02	-0.15 ± 0.04	-0.46 ± 0.23	-0.36 ± 0.10
***rpoS***	RNA polymerase sigma 38 subunit		-0.18 ± 0.22	-0.48 ± 0.05	0.36 ± 0.17	-0.18 ± 0.32
***ihfA***	Integration host factor, α subunit		0.31 ± 0.19	-0.46 ± 0.3	-0.15 ± 0.06	-0.59 ± 0.08
***crp***	cAMP repression protein		0.04 ± 0.15	0.31 ± 0.11	-0.22 ± 0.25	-0.05 ± 0.04
***pdhR***	Pyruvate dehydrogenase complex regulator		-0.70 ± 0.15	-1.11 ± 0.15	*n.d*.	*n.d*.
***fruR***	cAMP independent protein		-0.19 ± 0.01	-0.20 ± 0.16	-0.59 ± 0.06	-0.05 ± 0.04

***zwf***	Glucose 6-phosphate dehydrogenase	Pentose phosphate pathway	-0.04 ± 0.02	-0.05 ± 0.03	-0.39 ± 0.06	-0.24 ± 0.04

***ptsG***	Enzyme II^glc^, PTS subunit	Glucose transport	0.21 ± 0.15	-0.20 ± 0.16	*n.d*.	*n.d*.

***pykF***	Pyruvate kinase F	Glycolysis	-0.12 ± 0.08	0.26 ± 0.11	-0.85 ± 0.22	-0.88 ± 0.24
***pykA***	Pyruvate kinase A		-0.25 ± 0.16	0.67 ± 0.11	-0.83 ± 0.10	-0.13 ± 0.06
***aceE***	Pyruvate dehydrogenase		-0.07 ± 0.12	0.00 ± 0.05	-1.04 ± 0.18	-0.16 ± 0.07

***ldhA***	NAD dependent D-Lactate dehydrogenase	Fermentation pathways	-0.02 ± 0.11	0.73 ± 0.18	*n.d*.	*n.d*.
***dld***	NAD independent Lactate dehydrogenase		0.31 ± 0.26	-0.66 ± 0.05	*n.d*.	*n.d*.
***lldD***	NAD independent L-lactate dehydrogenase		-0.09 ± 0.12	-0.41 ± 0.03	*n.d*.	*n.d*.
***adhE***	Alcohol dehydrogenase		-0.76 ± 0.04	0.21 ± 0.04	*n.d*.	*n.d*.
***pflA***	Pyruvate formate-lyase activating enzyme		-0.09 ± 0.19	1.10 ± 0.10	*n.d*.	*n.d*.

***acs***	Acetyl-CoA synthethase	Acetate metabolism	0.37 ± 0.10	-0.71 ± 0.01	-1.28 ± 0.39	-1.36 ± 0.77
***poxB***	Pyruvate oxidase		-0.94 ± 0.30	-0.40 ± 0.05	-0.82 ± 0.10	-1.00 ± 0.11
***actP***	Acetate Permease		*n.d*.	*n.d*.	-0.28 ± 0.10	-1.13 ± 0.03
***ackA***	Acetate Kinase		*n.d*.	*n.d*.	-0.10 ± 0.01	-0.44 ± 0.00

***icdA***	Isocitrate dehydrogenase	TCA cycle	0.27 ± 0.04	-0.30 ± 0.16	-0.82 ± 0.17	0.31 ± 0.47
***sucA***	2-ketoglutarate dehydrogenase subunit		-0.12 ± 0.19	0.05 ± 0.03	-0.79 ± 0.17	-0.57 ± 0.36
***sdhC***	succinate dehydrogenase membrane protein		-0.14 ± 0.11	-0.77 ± 0.18	0.43 ± 0.58	-0.13 ± 0.47
***mdh***	Malate dehydrogenase		0.15 ± 0.03	-0.28 ± 0.08	-0.87 ± 0.08	-0.52 ± 0.17

***aceA***	Isocitrate lyase	Glyoxylate shunt	0.27 ± 0.06	-1.18 ± 0.09	-0.35 ± 0.25	-0.54 ± 0.23
***aceB***	Malate synthase		0.01 ± 0.11	-0.85 ± 0.04	-0.83 ± 0.15	-0.31 ± 0.31

***maeB***	Malate dehydrogenase (NADP^+^-requiring)	Glucogenogenesis/anaplerosis	0.25 ± 0.09	-0.26 ± 0.01	-0.39 ± 0.27	0.27 ± 0.34
***sfcA/maeA***	Malate dehydrogenase (NAD^+^-requiring)		0.26 ± 0.04	-0.18 ± 0.03	-0.05 ± 0.23	-0.45 ± 0.28
***pck***	Phosphoenolpyruvate carboxykinase		0.32 ± 0.07	1.04 ± 0.03	-0.57 ± 0.19	0.36 ± 0.13
***ppc***	Phosphoenolpyruvate carboxylase		-0.07 ± 0.08	0.68 ± 0.01	0.21 ± 0.25	0.29 ± 0.03
***pps***	Phosphoenolpyruvate synthase		1.27 ± 0.07	0.64 ± 0.00	-0.39 ± 0.05	-0.07 ± 0.37

Logarithmic ratios were determined using the ΔΔCt method. The *E. coli *BW25113 (wild type) strain was used as control under each growth condition. Experimental conditions are expressed in Materials and Methods section. (*n.d*. - not determined).

At the early exponential phase, the down regulation of *adhE *and *poxB *explained the lower production of ethanol and acetate in the mutant strain. With respect to glycolysis, the genes analyzed showed similar expression to the parent strain, while gluconeogenesis and anaplerosis were activated in the *pta *mutant.

At the onset of the stationary phase, the wild type and the *pta *mutant strains began to adapt to consume the acetate and lactate produced, respectively, after glucose exhaustion. Accordingly, in the *pta *mutant, gluconeogenic and some glycolytic genes (*pykA *and *pykF*) were activated. In this stage, several changes were also observed in genes related to the fermentation pathways. Surprisingly, although *pflA *was up-regulated, less formate was produced by the mutant strain. The genes related to lactate metabolism also presented a different expression pattern: *ldhA *was up-regulated while *lldD *and *dld *were down-regulated. The TCA cycle and the glyoxylate shunt were down-regulated in the mutant strain as well as the *acs *gene, which reflects the substantial differences in the pathways involved in the consumption of acetate (wild type strain) and lactate (*pta *mutant) in this phase.

In addition to these alterations, a few transcriptional regulators were also altered in both strains, reflecting the role of general control mechanisms in the alterations observed in the mutant. In fact, the regulation of *ihfA*, *cra*, *rpoS *and *pdhR *reflected these alterations, especially at the onset of the stationary phase.

### Acetate as the carbon source in aerobic batch cultures

The three *E. coli *strains were grown in acetate minimal medium in an attempt to understand the relevance of the Acs and Pta-Ack pathways in the assimilation of acetate and their relation to central metabolism.

#### Kinetics of cell growth and metabolism

**Figure 3:**
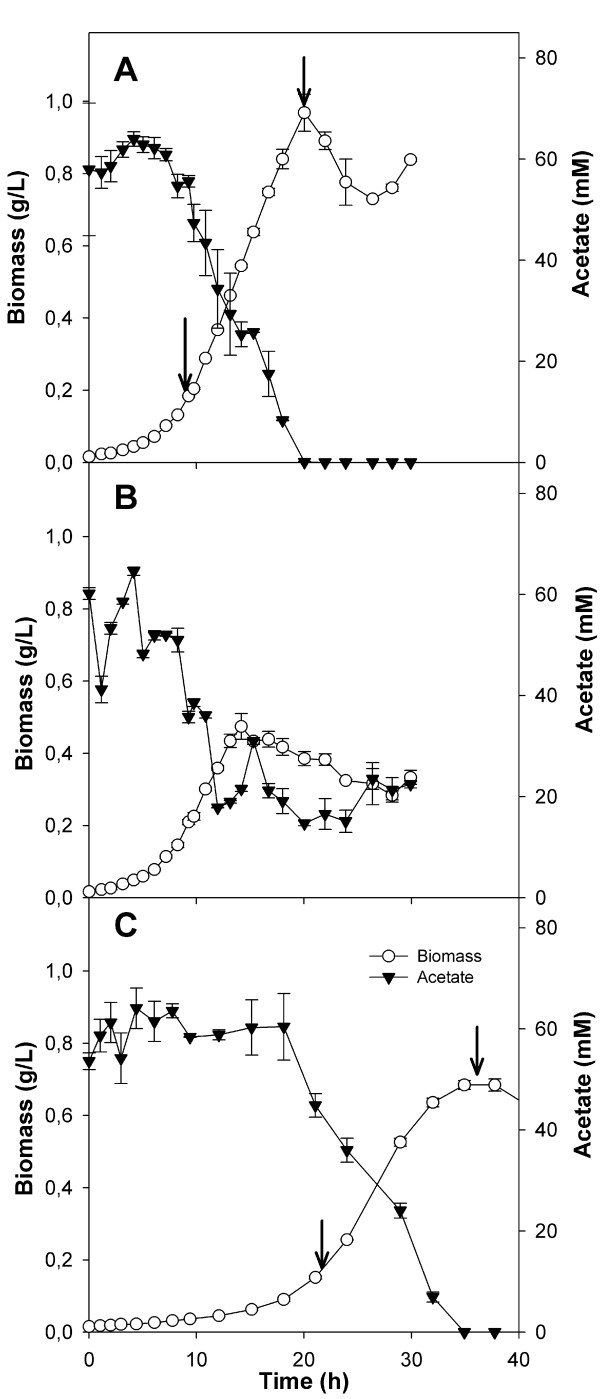
**Growth and metabolite production of A) *E. coli *BW25113 (wild type) and its B) Δ*acs *and C) Δ*pta *knockout derivative strains**. Experiments were performed using acetate as the carbon source. Sampling times for enzyme activities and/or gene expression are indicated by arrows. Assays were carried out as indicated in the Materials and Methods section.

The deletions greatly decreased the efficiency of acetate assimilation in both strains. The growth rate of the Δ*pta *strain in the acetate culture was almost half, compared to the control, while biomass yield was reduced in both mutant strains (Table 6). The acetate consumption rate in the Δ*pta *mutant was half that of the wild type strain, while in the Δ*acs *mutant it was slightly higher than in the control. However, the *acs *mutant strain was unable to fully consume the carbon source in the medium, probably as a consequence of the reversibility and low affinity of the Pta-Ack pathway [11]. No relevant metabolite production was assessed during culture and only traces of ethanol were detected (Figure 3).

**Table 6:** Growth and metabolic parameters of *E. coli *BW25113 (control), Δ*acs *and Δ*pta *mutant strains in batch aerobic cultures in acetate minimal medium.

**Growth parameters**	**BW25113**	Δ***pta***	Δ***acs***
**Biomass yield (g·g^-1^)**	0.26 ± 0.01	0.18 ± 0.01	0.13 ± 0.01
**Growth rate (h^-1^)**	0.28 ± 0.03	0.17 ± 0.02	0.27 ± 0.01
**Acetate uptake (mmol·(g·h)^-1^)**	24.67 ± 1.59	12.25 ± 0.29	29.10 ± 0.51

Culture conditions are expressed in the Materials and Methods section.

### Enzyme activities

Key enzyme activities were also studied during the early exponential phase of growth and at the onset of the stationary phase in the Δ*pta *mutant and control strains in acetate cultures (Table 4). Enzymes related to energy metabolism (glycolysis and TCA cycle) and the glyoxylate shunt showed altered levels. In fact, Pdh, Icdh and Icl activities were lower in the Δ*pta *strain, while Pyk activity remained steady. A similar Icdh/Icl ratio was observed for both strains in the exponential phase, indicating that the ability to direct acetyl-CoA towards biosynthetic pathways was not altered, while in the late exponential phase, the relative activity of the glyoxylate shunt in the Δ*pta *mutant was enhanced (Table 4). Moreover, the pentose phosphate shunt enzyme, Zwf, presented a higher activity in the mutant, generating biosynthetic intermediates and NADPH.

PoxB and Acs showed a surprising profile since the latter (which is the only feasible pathway for acetate assimilation in the Δ*pta *mutant) decreased in this strain, especially during the exponential phase, while PoxB (which excretes acetate to the medium) showed much higher activity, especially in the early exponential phase.

#### Gene expression by qRT-PCR

The expression level of 24 genes related to the central metabolism of *E. coli *was analyzed in the wild type and Δ*pta *strains. In general, the transcription of the genes analyzed was down-regulated within the different functional groups. At the early exponential phase, glycolysis, TCA cycle and glyoxylate shunt related genes were down-regulated, as well as those related to anaplerosis and gluconeogenesis (Table 5). Interestingly, the expression of *acs, actP *and *aceA*/*aceB *was down-regulated in the *pta *mutant, which supports the low growth rate observed in this strain (Table 5). At the onset of the stationary phase, fewer genes were down-regulated probably because of the adaptation of the *pta *mutant to the medium; *pykF*, *acs *and *poxB *showed substantially decreased expression.

Among regulatory genes, a noticeable down-regulation of the global regulator *cra *was observed during the exponential phase which could explain the down regulation of the GS, TCA cycle, and gluconeogenesis related genes. Moreover, a slight increase in *rpoS *expression, related to stress conditions, was observed in the same stage.

### Glucose anaerobic batch cultures

Finally, since acetate is the major product during anaerobic growth, *E. coli *BW25113 and the mutant strains were studied under anaerobiosis in glucose minimal medium.

#### Kinetics of cell growth and metabolites

**Figure 4:**
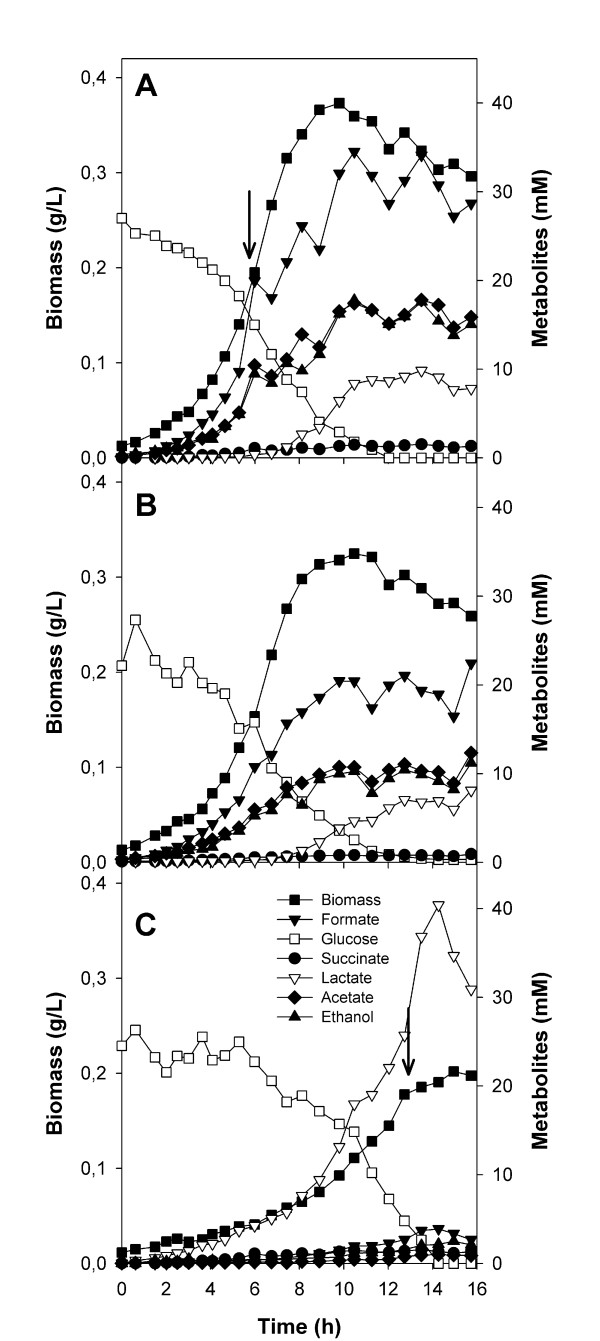
**Growth and metabolite production of A) *E. coli *BW25113 (wild type) and its B) Δ*acs *and C) Δ*pta *knockout derivative strains**. Experiments were performed using glucose as the carbon source and anaerobiosis. Sampling times for enzyme activities and/or gene expression are indicated by arrows. Assays were carried out as indicated in the Materials and Methods section.

Results indicate that the Δ*pta *strain grew at a lower rate and with a lower biomass yield than the control (Table 7). In addition, a lag phase was observed in the growth of this mutant (Figure 4). Similarly to that observed in the aerobic conditions, acetate, ethanol and formate were produced at lower rates than in the wild type strain, the main by-product being lactate. On the other hand, the Δ*acs *mutant showed a similar growth rate and metabolite production profile to the control.

**Table 7:** Growth and metabolic parameters of *E. coli *BW25113 (control) Δ*acs *and Δ*pta *mutant strains in batch anaerobic cultures in glucose minimal medium.

**Growth parameters**	**BW25113**	**Δ*pta***	**Δ*acs***
**Biomass yield (g·g^-1^)**	0.10 ± 0.003	0.05 ± 0.003	0.09 ± 0.003
**Growth rate (h^-1^)**	0.48 ± 0.03	0.22 ± 0.02	0.43 ± 0.02
**Glucose uptake rate [mmol·(g·h)^-1^]**	25.40 ± 0.96	29.16 ± 0.15	25.36 ± 1.11
**Acetate production rate [mmol·(g·h)^-1^]**	19.15 ± 1.65	0.79 ± 0.17	16.15 ± 2.09
**Ethanol production rate [mmol·(g·h)^-1^]**	17.13 ± 3.14	2.00 ± 0.39	15.72 ± 2.13
**Formate production rate [mmol·(g·h)^-1^]**	37.34 ± 3.14	4.27 ± 0.64	31.00 ± 3.73
**Lactate production rate [mmol·(g·h)^-1^]**	0.91 ± 0.05	40.51 ± 5.53	0.30 ± 0.01
**Succinate production rate [mmol·(g·h)^-1^]**	2.19 ± 0.46	1.31 ± 0.22	1.33 ± 0.15

Culture conditions are expressed in the Materials and Methods section.

#### Energetic and redox state

The intracellular ATP levels and the redox state of the Δ*pta *strain were measured at the exponential phase of growth in anaerobic batch cultures. The Δ*pta *mutant exhibited significantly higher ATP levels than the control strain, while the redox state was almost the same in both strains (Table 3).

#### Enzyme activities

The greatest differences observed in enzyme activities affected energy metabolism in response to the anaerobic conditions: the Icdh, Mdh and Pdh activities were down-regulated and the glycolysis (Pyk), pentoses phosphate pathway (Zwf) and fermentative pathways (Pta and Acs) were up-regulated (Table 4). The differences found between the Δ*pta *and the control strains were similar to those found in glucose aerobic batch cultures. The pentose shunt Zwf showed very low activity in the Δ*pta *mutant, reflecting the lower growth-associated biosynthetic activity. As demonstrated in the glucose aerobic cultures, Pdh also had higher activity in the Δ*pta*. The GS and acetate metabolism were also affected: in the knockout mutant Icl and Pox presented lower activity and almost no Acs activity was detected. It should be underlined that, under anaerobic conditions, both Pox and Acs activities were high (Table 4), meaning that i) Pta-Ack is not the sole acetate-producing pathway in *E. coli *and ii) the co-expression of Pox and Acs pathways could lead to an acetate-consuming cycle with lower energetic efficiency, as previously suggested by Flores et al[23].

## Discussion

In this work, further insight into the interrelations between the acetyl-CoA/acetate pathways (Pta-Ack and Acs) and the central metabolism of *E. coli *is presented. The effect of the deletion of *pta *and *acs *was evaluated in three different scenarios: growth on glucose (aerobic and anaerobic conditions) and on acetate (aerobic conditions). While *acs *deletion had only slight effects on bacterial physiology and metabolism, the deletion of *pta *provoked a strong perturbation, indicating its great importance. For that reason and to further understand how this mutation affects *E. coli*, a detailed characterization of this mutant was performed using a multilevel analysis approach (gene expression, enzyme activities and metabolic rates). Growth on different carbon sources further underlined the role of *pta*, while the comparison of aerobic/anaerobic cultures yielded less valuable information, probably because *pta*, as is known, is constitutively expressed under both conditions [5,11]. Thus, aerobic and anaerobic glucose cultures will be jointly discussed and compared to acetate cultures.

It has been previously described that when *pta *is deleted in *E. coli*, pyruvate accumulates in the cell [24,25], as a result of which the fermentation profile is completely altered. In our work, almost no acetate or ethanol was produced in aerobic or anaerobic glucose cultures, being substituted by lactate (Figures 2 and 4). It is well known that Ldh of *E. coli *has a high K_m _(low affinity) towards pyruvate, and lactate production is only observed when pyruvate accumulates [26]. Thus, the *pta *strain also suffered an overflow, with lactate being produced instead of acetate and ethanol, in order to restore the NADH/NAD^+ ^balance and allow for continued glycolysis (Table 2). Contrary to what has previously been proposed [24], lactate production was not due to the increased expression of lactate dehydrogenase (*ldhA*), but rather to pyruvate accumulation [25]. In fact, the fermentation pathways genes analyzed here, as well as *poxB *(acetate metabolism), were down-regulated in the *pta *mutant during the exponential phase, with the exception of *dld*, which codes for a NAD^+^-independent lactate dehydrogenase. Interestingly, with entry into the stationary phase, the expression pattern of *ldhA *and *dld *(coding for NAD^+^-dependent and NAD^+^-independent lactate dehydrogenases) was inverted, which explains why, in the stationary phase, the *pta *mutant recycled the previously excreted lactate, resulting in diauxic growth and increased biomass yield (Figure 2, Table 5).

**Figure 5:**
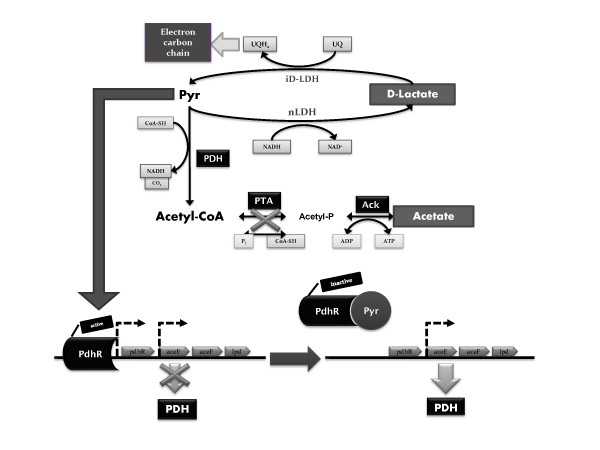
**Simplified model for the alterations in the metabolic network of *E. coli *after *pta *deletion**. Lactate is produced as a result of the knock-out of the *pta *gene and it is used to produce ATP by the combined action of lactate dehydrogenases, nLDH and iDLDH. Moreover, pyruvate accumulation would activate the *pdh *operon. The enzymes involved are shown in the figure [ACK (*ackA*), acetate kinase; PTA (*pta*), phosphotransacetylase, PDH (*aceEF;lpd*) pyruvate dehydrogenase (Ecocyc-Metacyc [45]).

The deletion of *pta *caused a further metabolic rearrangement. The analysis of genes and enzyme activities related to the central metabolism threw more light on these alterations, most of which were caused by pyruvate/acetyl-CoA accumulation. In general, data on relative gene expression, enzyme activities and metabolic fluxes fitted well. However, it is well known that multiple levels of regulation exist in the cells, which can lead to non-conclusive results. This is the case of the Pdh complex, which is coded by three structural genes (*aceEF *and *lpdA*) and a regulator (*pdhR*) included within the same operon. This operon is subjected to complex transcriptional regulation, which involves a specific regulator (PdhR) and three different promoters [27,28]. The activity of Pdh is subjected to tight regulation by NADH [29] and acetyl-CoA [30]. The accumulation of acetyl-CoA would inhibit the Pdh complex, resulting in the accumulation of pyruvate. This is known to prevent the binding of PdhR to the promoter of the *pdh *operon (Figure 5; [31]), finally leading to the expression of *aceEF *and *lpdA*. Consistent with this regulatory scheme, a ten-fold decrease in the *pdhR *transcript level was observed in the *pta *mutant in glucose cultures, while no difference in that of *aceE *was observed (Table 5). Furthermore, the Pdh activity in the *pta *mutant was double that of the wild type strain (Table 4), which underlines the feasibility of post-transcriptional and post-traductional control mechanisms, which have not been described so far in *E. coli*.

In glucose culture, the TCA cycle genes, *icdA *and *mdh *(and the corresponding enzymes Icdh and Mdh), were up-regulated (Tables 4 and 5). Pyruvate also acts on the phosphatase and kinase activities of AceK (which controls Icdh activity by covalent modification) and thus, pyruvate accumulation would result in increased Icdh activity [32]. Moreover, IclR (the repressor of the GS) is also activated by pyruvate [33], resulting in *aceBA *down-regulation. In fact, *aceBA *was repressed in the stationary phase on glucose and in both phases on acetate, and lower Icl activity was detected in all phases. Therefore, pyruvate accumulation would seem to increase TCA cycle fluxes by modulating the two activities which control the isocitrate node.

Finally, the expression of phosphoenolpyruvate synthase (Pps) is also activated by pyruvate and lactate [34], controlling the flux through gluconeogenesis and altering the metabolic yields in *E. coli*. In fact, *pps *and *pck *were highly activated in the glucose cultures. This increase in the gluconeogenic pathways could be a means to compensate the down-regulation of the GS and would explain the excellent growth characteristics of this mutant in aerobic glucose cultures.

Enzyme activities analyzed during anaerobic cultures mostly reflected the effect of the absence of oxygen. In general, the rearrangements observed in the *pta *mutant strain in the aerobic and anaerobic conditions were similar.

In the acetate cultures, both Pta and Acs activities were at a high level in the wild type strain, and the Pta/Acs ratio was lower than on glucose, indicating the induction of Acs (Table 4). The expression of *acs *is induced by acetate [35], as verified in the wild type strain in the stationary phase on glucose and in both phases on acetate. Acetate production/assimilation pathways were greatly altered in the *pta *mutant strain. Both *acs *and *poxB *genes were repressed in the *pta *mutant strain in glucose and acetate cultures. Accordingly, Acs activity was much lower in all the conditions assayed (Table 4), a phenomenon already reported [35]. The down-regulation of *poxB *in the *pta *mutant would allow increasing growth yield in acetate as carbon source. However, in the *pta *mutant i) in glucose cultures, PoxB activity was similar to that of the wild type and, ii) in acetate, it reached values higher than those observed in glucose cultures or by the wild type strain in acetate cultures (Table 4). This implies the occurrence of a potential futile cycle *in vivo*, which would lead to decreased energetic efficiency in carbon assimilation. In fact, the biomass yield in acetate cultures decreased by 40% in the *pta *mutant strain compared to that observed in the glucose cultures (Tables 2 and 6). Some authors have proposed that the expression of *poxB *under conditions mimicking carbon starvation might be seen as a carbon scavenging response leading to increased biomass yield upon Pdh (aerobic) or Pfl (anaerobic) repression [36]. The PoxB/Acs pathway is a bypass of these routes and all the three involve the formation of a CO_2 _molecule. When the PoxB/Acs, the glyoxylate shunt, the TCA cycle and/or the PEP-glyoxylate [37] pathways are jointly considered, the overall balance of the route changes in terms of energy and redox efficiency. Compared with the TCA cycle, the PEP-glyoxylate pathway allows to skip the formation of one GTP and NADPH per PEP molecule. On the other hand, compared with the Pdh route, the metabolization of pyruvate through the PoxB/Acs pathway involves the net consumption of ATP and the formation of ubiquinol (instead of NADH). Interestingly, both PoxB/Acs and the PEP-glyoxylate cycle are related to hunger and/or slow-growth conditions [37,38]. Thus, under slow growth conditions, their joint expression can lead to lower production of (i) ATP/GTP and (ii) NADPH and (iii) formation of ubiquinol. This means that in slow growing bacteria the metabolism of cells is switched towards sub-optimal efficiency. This may allow a proper coupling of the production and consumption of energy and reducing power or maybe it is simply the price the bacteria have to pay to be able to adapt to variable stress conditions. This switch to lower efficiency is physiological and crucial during the down-regulation of other pathways, since *poxB *deletion leads to inefficient growth, especially in some genetic backgrounds (such as *pts*^- ^strains) [36,38].

The deletions strongly decreased the efficiency of acetate assimilation and growth, indicating that the Pta-Ack and the Acs pathways must act together. This was especially relevant in the case of the *pta *mutant strain, probably as a result of the specific regulation and characteristics of Acs. The lag observed in the growth of this mutant was probably related to the repression of Acs in the exponential phase (since it is especially related to the stationary phase) [35] in the *pta *mutant. On the other hand, the Pta-Ack pathway alone does not allow for an efficient use of acetate as carbon source because of i) its reversibility and ii) lower affinity towards the substrate (K_m _7-10 mM, [39]). However, other non-identified factors cannot be ruled out.

Metabolic pathways had to rearrange towards a more efficient use of acetate as carbon source in the *pta *mutant strain. In these cultures, the expression of almost all the genes assayed decreased. A down-regulation of Acs, glycolysis, TCA cycle and GS was observed in the *pta *mutant strain. However, the GS, which is essential for growth on acetate, showed high activity in acetate cultures in both strains, with levels of the same order of magnitude as those of Icdh (Table 4). Further, the Icdh/Icl ratio was similar in both strains during the exponential phase and sharply decreased during the stationary phase in the case of the *pta *strain, revealing the adaptation of this mutant to acetate culture (Table 4). Surprisingly, *pta *deletion caused a decrease in Acs expression and activity, which partially explains the low growth and acetate uptake rates and biomass yield observed (Table 6).

These evident effects could stem from changes in the transcriptional regulators of the cellular metabolism. It could be hypothesized that the accumulation of acetyl-phosphate could be responsible for the poor growth characteristics of the *pta *mutant. However, both *ackA *and *ackA-pta *deletion mutants showed similarly affected growth in acetate medium even though no acetyl-phosphate accumulation was detected [40,41]. Among the regulators analyzed, the down-regulation of *cra *(*fruR*) was the most dramatic change observed, revealing that the mutation in *pta *had a global effect on cellular physiology. In fact, Cra regulates the expression of a number of genes from glycolysis, TCA cycle, GS and gluconeogenesis and plays an important role in the control of carbon fluxes [42-45]. Moreover, RpoD (σ^70^) and RpoS (σ^S^) are sigma subunits of RNA polymerase and are differentially expressed in the exponential and stationary phases of growth. The slight up-regulation of *rpoS *in the exponential phase of growth on acetate, allows us to ascertain why cell growth was so affected, since stress conditions severely compromised cellular physiology. Microarray studies have indicated that the expression of several important genes of the acetate metabolism such as *acs*, *aceAB*, *cysDEK*, *fadR*, etc, are significantly affected by *rpoS *[46]. During the late exponential phase of the acetate cultures, changes in gene expression were not as noticeable as in the exponential phase. In fact, the levels of expression of the different transcriptional regulators analyzed were similar in the wild type and *pta *mutant strains, including *rpoS *and *cra*. In this case, *ihfA *(coding for the integration host factor α subunit), a transcription factor which is relevant in the stationary phase and in the metabolism of acetate, was strongly down-regulated, probably causing the repression of the GS, TCA cycle and acetate metabolism. Moreover, IHF exerts its effect on acetate, while hardly any effect is observed on glucose [47].

Oh et al. [48] analyzed the transcriptional response of *E. coli *to growth on acetate compared to glucose as carbon source, finding that in the presence of acetate most of the altered genes were down-regulated, especially those involved in the cellular machinery. The authors related this to the slow-down in cellular growth and the less active metabolism. These authors also underlined the importance of Acs, GS and gluconeogenesis. Identical responses to acetate were observed in this work for the control strain (data not shown) and even more drastic responses to acetate were determined for the *pta *mutant. In fact, although Acs is the main acetate uptake pathway, it is recognized that Pta-Ack increases acetate influx [48].

Contrary to what has previously been described [49], we demonstrate herein that *pta *is not essential for anaerobic growth, although its deletion leads to inefficient growth. Moreover, the mutation of *pta *also affected the redox and energy state of *E. coli *(Table 3). Once the aerobic glucose cultures reached the stationary phase, the redox environment of the *pta *mutant strain was more reduced than that of the wild type strain (while the amount of intracellular ATP was higher), which reflects lactate consumption. Similarly, a more reduced environment was also observed in the exponential phase of the anaerobic glucose culture, suggesting that the Pta-Ack pathway is also important for ensuring a proper redox balance, as further substantiated by the slow growth.

On the other hand, analysis of the metabolism of the *pta *mutant underlines the importance of pyruvate as mediator of the metabolic alterations observed, where it acts not only as an intermediate of the central metabolism but also as an allosteric regulator of several enzymes and activator/repressor of transcription factors. Pyruvate is an allosteric activator of Ldh and the Pfl-activating enzyme, and also activates Pta, AceK (Icdh phosphatase/kinase) and IclR; on the other hand, it inhibits the Pdh complex, AceK and Pfl-deactivase. On top of this, the related PEP inhibits Icl, Ppc, Pck and regulates AceK [50]. Taken together, the results we present herein further support the close interconnection between the metabolisms of acetate and isocitrate, with pyruvate and PEP as feasible actuators. In fact, the PEP-pyruvate-OAA node is composed of enzymes linking gluconeogenesis with the TCA cycle and the GS, allowing anaplerosis [51], and these pathways were up-regulated in glucose cultures. Moreover, under these conditions, both Pyk isozymes were down-regulated, while Pps, which catalyzes the first step of the gluconeogenic pathway, was up-regulated. In acetate cultures, only Ppc was slightly up-regulated in the *pta *mutant. On acetate, the gluconeogenic pathway, Pck or the malic enzymes need to be active [48] and, in fact, these were repressed (Table 5). Additionally, this reveals that the PEP-pyruvate-OAA node acts as a bottleneck for the *pta *mutant when growing on acetate and *E. coli *rearranges flux distributions around the PEP-glyoxylate cycle.

It is likely that the constitutive expression of Pta allows the rapid adaptation and survival of *E. coli *in changing environments. Acetate metabolism has long been regarded as crucial for the use of *E. coli *as cellular factory. Overflow metabolism leads to decreased carbon yield, growth and protein production inhibition [2,18,19]. The present work provides a further insight into the acetyl-CoA/acetate metabolic node of *E. coli*. Lactate overflow in the *pta *mutant strain shows that metabolic overflow is necessary for adequate metabolic balancing. However, both *pta *and *acs *mutations severely affected the ability of *E. coli *to adapt to changes in their environment (such as the use of acetate as carbon source and/or anaerobiosis). Further work is necessary in order to evaluate the implications of these mutations for the improvement of *E. coli *as a cellular factory. At present, our group is working on the design of strain engineering strategies based on these pathways and to unravel the intricate regulatory networks underlying these observed mechanisms.

## Conclusion

The activity of the central pathways of E. coli is affected by the deletion of the genes of the acetyl-CoA/acetate metabolism. The mutations alter the co-regulation of the acetate metabolism, glyoxylate shunt and the anaplerotic/gluconeogenic pathways, affecting the efficient assimilation of the carbon sources. The reversibility and low-affinity of the Pta-Ack pathway resulted in the low efficiency of acetate consumption following acs deletion, while pta deletion severely compromised the adaptation capacity of E. coli to anaerobic conditions or to the use of acetate as carbon source. On the other hand, in the Δpta strain, the metabolism had to rearrange in order to buffer the ATP and NADH/NAD^+ ^pools. The production of lactate and, especially, the altered regulation of the Pdh complex in the Δpta strain reflects that i) metabolic overflow is a crucial mechanism to ensure continued glycolytic activity, ii) the metabolic pools are altered as a consequence of gene deletions and iii) metabolic fluxes can be rearranged in order to ensure redox homeostasis of the cell. Finally, the activation of PoxB in acetate cultures was detected.

Moreover, it is demonstrated that pta is not essential for anaerobic growth, although its deletion drastically decreases growth efficiency under certain conditions, underlining that these pathways, and the PEP-pyruvate-OAA node are highly relevant for ensuring the adaptability of this bacterium to environmental changes and for its use in bioprocesses.

## Methods

### Bacterial strains and cultures

*E. coli *BW25113 strains (wild-type and *acs *and *pta *deletion mutants) were used throughout this study (Table 1). The *E. coli *BW25113 derivatives carry complete gene deletions and belong to the KO-collection (; [52]). The standard minimal media (pH 7.4) contained: 2.6 g/L (NH_4_)_2_SO_4_, 1.0 g/L NH_4_Cl, 0.5 g/L NaCl, 15.0 g/L Na_2_HPO_4_·12 H_2_O, 3.0 g/L KH_2_PO_4_, 50.0 mg/L FeCl_3_·6 H_2_O, 65.0 mg/L EDTA Na_2_, 1.8 mg/L ZnSO_4_·7 H_2_O, 1.8 mg/L CuSO_4_·5 H_2_O, 1.2 mg/L MnSO_4_·H_2_O, 1.8 mg/L CoCl_2_·6 H_2_O, 2.0 mM MgSO_4_, 0.2 mM CaCl_2_, and 0.3 μM thiamine·HCl. As carbon source, 20 mM glucose or 60 mM acetate were used. Aerobic 200 mL batch cultures were grown in 1 L flasks at 37°C on a rotary shaker at 150 rpm. Frozen 20% glycerol stock cultures were used to inoculate glucose-supplemented minimal media precultures. Cultures were inoculated to an optical density (OD_600 nm_) of 0.05 with exponentially growing precultures.

### Analytical procedures

To estimate cell concentration, cells were resuspended in 65 mM phosphate buffer pH 7.5 and absorbance was measured at 600 nm (Pharmacia Biotech Novaspec II Spectrophotometer, Uppsala, Sweden). A_600 _values and dry cell weight were correlated for each strain.

Extracellular metabolites (acetate, formate, ethanol, succinate, pyruvate and lactate) were analyzed by HPLC (Shimadzu Scientific Instruments, Columbia, MD), equipped with differential refractive (Shimadzu Scientific Instruments, Columbia, MD) and UV (Waters, Milford, MA) detectors, using a cation-exchange column (HPX-87H, BioRad Labs, Hercules, CA). The mobile phase was 5 mM H_2_SO_4 _at 0.4 ml·min^-1 ^flow rate and 65°C. Glucose was assayed by a glucose (HK) assay kit (Sigma Aldrich, Saint Louis, MO) according to the manufacturer's recommendations. Measurements were performed in a Microplate Spectrophotometer Synergy HT (Bio-Tek, Winooski, VT).

### Preparation of RNA and RT-PCR

Total RNA was isolated from 3·10^8 ^cells by Qiagen Rneasy^® ^Mini Kit (QIAGEN Ibérica, Madrid, Spain) according to the manufacturer's recommendations. Additionally, Dnase I digestion of the isolated RNA was performed using the Rnase-Free Dnase Set (QIAGEN Ibérica, Madrid, Spain) to avoid DNA interferences during PCR steps. Isolated RNA purity and concentration were assessed in a NanoDrop^® ^ND-1000 spectrophotometer (NanoDrop Technologies, Wilmington, DE). RNA quality was evaluated by microfluidic capillary electrophoresis on an Agilent 2100 Bioanalyzer (Agilent Technologies, Palo Alto, CA) using Agilent RNA 6000 Pico kit. Chips were prepared and loaded according to the manufacturer's instructions. Isolated RNA was stored at -80°C for no longer than three days.

One microgram of high quality RNA (rRNA ratio [23S/16S] ≈ 1.6, RNA integrity number [RIN] > 9.0, and A^260^/A^280 ^ratio > 2.0) was reverse transcribed with TaqMan^® ^Reverse Transcription Reagents (Applied Biosystems, Foster City, CA) according to the manufacturer's protocol and stored at -20°C prior to use. Briefly, a 50 μL reaction mixture was incubated in a Peltier Thermal Cycler 200 (MJ Research Inc., Boston, MA) for 10 min at 25°C, 30 min at 48°C and 5 min at 95°C.

The primers used in this work (see Additional file 1) were designed using the Primer Express^® ^Software v3.0 (Applied Biosystems, Foster City, CA) and ordered from Applied Biosystems (Cheshire, UK). The *polA*, *dnaA *and *rrsA *genes (encoding DNA polymerase I, transcriptional dual regulator and 16S ribosomal RNA, respectively) were used as internal control for relative quantification.

Quantitative PCR was performed in a 7300 Real-Time PCR System (Applied Biosystems, Foster City, CA) using Power SYBR^® ^Green PCR Master Mix (Applied Biosystems, Foster City, CA) according to the manufacturer's instructions. Briefly, 50 μL reactions mixtures, with 10 ng template cDNA and 15 pmol of each primer, were incubated for 2 min at 50°C, 10 min at 95°C and 40 PCR cycles (15 s at 95°C and 1 min at 60°C). An additional dissociation step (15 s at 95°C, 30 s at 60°C and 15 s at 95°C) was added to assess non-specific amplification. PCRs were run in triplicate. Raw data were transformed into threshold cycle (C_t_) values. Relative gene expression for each mutant, compared to wild type, was calculated by the comparative C_t _Method (ΔΔC_t_).

### Enzyme assays

The enzyme activity assays were optimized for the conditions and media. All measurements were carried out in a microplate spectrophotometer Synergy HT (Bio-Tek, Winooski, VT). Enzyme activity was defined as μmol of substrate consumed per minute and mg of protein (U/mg). All enzyme activities were measured at 37°C.

In each case, reactor bulk liquid samples were withdrawn and centrifuged at 16,000 × g at 4°C. The supernatant was removed and cells were resuspended in 65 mM phosphate buffer (pH 7.5). Cells were sonicated on ice for 3 cycles (20 s each), with a probe of 3 mm diameter of a Vibra Cell VC 375 ultrasonic processor (Sonics Materials, Danbury, CT). The extract was centrifuged for 15 min at 20,000 × g and 4°C to remove cell debris and the supernatant was used for subsequent activity measurements. Protein content was determined by the method of Lowry modified by Hartree [53].

#### Isocitrate dehydrogenase (Icdh)

The method was described by Aoshima et al. [54]. The measurement buffer was 65 mM potassium phosphate (pH 7.5). The reaction components were 5 mM MgCl_2_, 2 mM NADP^+ ^and 2.5 mM D, L-isocitrate. The enzyme activity was followed by the increase in NADPH absorbance at 340 nm (ε_NADPH _= 6.220 *M*^-1^cm^-1^). One unit of enzyme activity was that required for the generation of 1 μmol of NADPH per min.

#### Isocitrate lyase (Icl)

The assay was that described by Aoshima et al. [54], using the same buffer as above. The reaction mixture was composed of 5 mM MgCl_2_, 20 mM phenylhydrazine and 5 mM D, L-sodium isocitrate. The enzyme activity was followed by the increase in absorbance at 324 nm due to the reaction of the glyoxylate produced with phenylhydracine (ε_adduct _= 16,8 M^-1^cm^-1^). One unit of enzyme activity was taken as that needed to generate 1 μmol of adduct per min.

#### Acetyl-CoA synthetase (Acs)

The method used was that established by Lin et al. [55]. The measurement buffer was 100 mM Tris-HCl (pH 7.8). The reaction mixture contained 5 mM D, L-Malate, 1 mM ATP, 2.5 mM MgCl_2_, 0.1 mM coenzyme A, 3 mM NAD^+^, 2.5 U/mL malate dehydrogenase, 1.25 U/mL citrate synthase and 100 mM sodium acetate. The acetyl-CoA synthetase activity was followed as the increase in NADH absorbance at 340 nm (ε_NADH _= 6.220 M^-1^cm^-1^). Enzyme activity unit was defined as the enzyme generating 1 μmol of NADH per min.

#### Glucose 6-phosphate dehydrogenase (Zwf)

The method was that of Peng et al. [56]. The measurement buffer was Tris-HCl 100 mM (pH 7.5), and the reaction mixture contained 10 mM MgCl_2_, 1.5 mM NADP^+ ^and 10 mM glucose 6-phosphate. The enzyme activity was followed for 5 min as the increase in NADPH absorbance at 340 nm (ε_NADH _= 6.220 M^-1^cm^-1^), one unit being taken as the enzyme required to generate of 1 μmol of NADPH per min.

#### Phosphotransacetylase (Pta)

*T*he assay was carried out as in Peng et al. [56]. The measurement buffer was 250 mM Tris-HCl, pH 7.8. The reaction components were 1 mM MgCl_2_, 10 mM D, L-malic acid, 3 mM NAD^+^, 0.5 mM coenzyme A, 2.5 U/mL malate dehydrogenase, 1.25 U/mL citrate synthase and 10 mM acetyl-phosphate. The enzyme activity was followed as the increase in NADH absorbance at 340 nm (ε_NADPH _= 6.220 M^-1^cm^-1^), one unit being taken as the enzyme required for the generation of 1 μmol of NADH per min.

#### Pyruvate dehydrogenase complex (Pdh)

*T*he method was that of Brown et al. [57]. The measurement buffer was 50 mM potassium phosphate (pH 8.0), and the reaction components were 1 mM MgCl_2_, 0.5 mM thyamine pyrophosphate, 0.5 mM L-cysteine, 2.5 mM NAD^+^, 0.1 mM coenzyme A and 10 mM sodium pyruvate. The enzyme activity was followed as the increase in NADH absorbance at 340 nm (ε_NADH _= 6.220 M^-1^cm^-1^). One enzyme activity unit was taken to be the enzyme required to generate 1 μmol of NADH per min.

#### Malate dehydrogenase (Mdh)

The method was that of Park et al., [58]. The measurement buffer was 65 mM potassium phosphate (pH 7.5), with 0.5 mM NADH, and 0.2 mM oxaloacetic acid as substrates. The enzyme activity was followed as the decrease in NADH absorbance at 340 nm (ε_NADH _= 6.220 M^-1^cm^-1^), one unit being taken as the enzyme required for the consumption of 1 μmol of NADH per min.

#### Pyruvate oxydase (PoxB)

The method was that of Abdel-Hamid et al. [38] with minor modifications. The measurement buffer was 65 mM potassium phosphate buffer (pH 6.0), and the reaction mixture was 5 mM MgCl_2, _0.25 mM thyamine pyrophosphate, 2.5 mM potassium ferricyanide and 100 mM sodium pyruvate. The enzyme activity was followed for 10 min as the increase in potassium ferricyanide absorbance at 405 nm (ε_ferricyanide _= 0.093 *M*^-1^cm^-1^). One enzyme activity unit was taken as the enzyme generating 1 μmol of ferricyanide per min.

#### Pyruvate kinase (Pyk)

The method was that of Peng et al. [56] with minor modifications. The measurement buffer was 50 mM (pH 6.5) Bis-Tris buffer, and the reaction mixture was 25 mM MgCl_2_, 10 mM KCl, 0.25 mM Dithiotreitol, 0.5 mM NADH, 2.5 mM ADP, 2.5 U/ml L-lactic dehydrogenase and 5 mM phosphoenolpyruvate. The enzyme activity was followed as the decrease in NADH absorbance at 340 nm (ε_NADH _= 6.220 M^-1^cm^-1^), one unit being taken as the enzyme required for the consumption of 1 μmol of NADH per min.

### Determination of ATP content and NADH/NAD^+ ^ratio

The energy content *per *unit of cell was determined as the ATP level and NADH/NAD^+ ^ratio throughout the experiments. For ATP measurement, the HS II bioluminescence assay kit from Boëhringer (Mannhein, Germany), based on the luciferase enzyme using a microplate spectrophotometer Synergy HT (Bio-Tek, Winooski, VT) was used. DMSO was used for cell lysis. Cell content was determined assuming an intra-cellular volume of 1.63 μL/mg [59].

Reducing power, taken as the NADH/NAD^+ ^ratio, was determined as in Snoep et al. [60]. For the measurements, an enzymatic method based on alcohol dehydrogenase was used. The extraction of the reduced or the oxidized forms was carried out by two different methods, involving alkali or acid extraction.

## List of abbreviations used

*aceBAK*: glyoxylate shunt operon; *aceE*: subunit E1p of pyruvate dehydrogenase complex; *aceF*: lipoate acetyltransferase subunit of pyruvate dehydrogenase complex; Ack (*ack*): acetate kinase; Acs (*acs*): acetyl-CoA synthetase; ADH (*adhE*): alcohol dehydrogenase; cAMP: cyclic AMP; CoASH: coenzyme A; Cra/FruR (*cra*): catabolite repressor activator; Crp (*crp*): catabolic repressor via cAMP receptor protein; Icdh (*icd*): isocitrate dehydrogenase; IcIR (*iclR*): glyoxylate shunt repressor; Icl (*aceA*): isocitrate lyase; iD-Ldh (*dld*): D-lactate dehydrogenase (NAD^+^-independent); IHF α subunit (*ihfA*): subunit of integration host factor; iL-Ldh (*lldD*): L-lactate dehydrogenase (NAD^+^-independent); Ldh (*ldhA*): lactate dehydrogenase (NAD^+^-dependent); Lpd (*lpd*): lipoamide dehydrogenase, pyruvate dehydrogenase E3 monomer; MaeB (*maeB*): malic enzyme NADP^+^-requiring; Mdh (*mdh*): malate dehydrogenase; MS (*aceB*): malate synthase; OAA: oxaloacetate; Pcc (*ppc*): PEP carboxylase; Pck (*pck*): PEP carboxykinase; Pdh: pyruvate dehydrogenase complex; PdhR (*pdhR*): pyruvate dehydrogenase complex regulator; PEP: phosphoenolpyruvate; Pfl (*pflB*): pyruvate formate lyase; PflA (*pflA)*: pyruvate formate lyase activating enzyme; *polA*: DNA polymerase I; PoxB (*poxB*): pyruvate oxidase; Pps (*pps*): phosphoenolpyruvate synthase; Pta (*pta*): phosphotransacetylase; PtsG (*ptsG*): subunit of Enzyme II^glc^; Pyk (*pykF*, *pykA*): pyruvate kinase; RpoD (or σ^32^): sigma subunit of RNA polymerase; RpoS (or σ^S^): sigma subunit of RNA polymerase; Sdh (*sdhC)*: succinate dehydrogenase membrane protein; SfcA (*sfcA/maeA*): malic enzyme NAD^+^-requiring; *sucA*: subunit of E1 (0) component of 2-oxoglutarato dehydrogenase; TCA: tricarboxylic acids cycle; Zwf (*zwf*): glucose 6-phosphate dehydrogenase.

## Competing interests

The authors declare that they have no competing interests.

## Authors' contributions

MC, JLI and VB designed the project. SCC, JMP and SR implemented the experimental procedures. SCC and JMP designed and performed the experiments. SCC, VB and MC analyzed the data and drafted the manuscript. All authors read and approved the final version of the manuscript.

## Supplementary Material

Additional file 1 **Primers used for real time PCR**. The primers used in this work were designed using the Primer Express^® ^Software v3.0 (Applied Biosystems, Foster City, CA) and ordered from Applied Biosystems (Cheshire, UK). The *polA*, *dnaA *and *rrsA *genes (encoding DNA polymerase I, transcriptional dual regulator and 16S ribosomal RNA, respectively) were used as internal control for relative quantification. Click here for file
