# Supplementary material for: An insight into the role of phosphotransacetylase (pta) and the acetate/acetyl-CoA node in Escherichia coli
Source: Microb Cell Fact. 2009 Oct 24;8:54. doi: 10.1186/1475-2859-8-54 (PMC2774668; doi:10.1186/1475-2859-8-54)
Supplement: Additional file 1 — Primers used for real time PCR. The primers used in this work were designed using the Primer Express® Software v3.0 (Applied Biosystems, Foster City, CA) and ordered from Applied Biosystems (Cheshire, UK). The polA, dnaA and rrsA genes (encoding DNA polymerase I, transcriptional dual regulator and 16S ribosomal RNA, respectively) were used as internal control for relative quantification. [file 1475-2859-8-54-S1.DOC]

| **Group** | ***Gene*** | **Forward primer** | **Reverse primer** |
| --- | --- | --- | --- |
| **Internal control** | *polA* | 5´-GCTGAACGTGCAGCCATTAA-3´ | 5´-CAATCATCGCCCGTTTGATAA-3´ |
|  | *dnaA* | 5´-TGGCGAAAGAGCTGACTAACC-3´ | 5´-ACGGCAGGCATGAAGCA-3´ |
|  | *rrsA* | 5´-CCTTACGACCAGGGCTACACA-3´ | 5´-CACTTTATGAGGTCCGCTTGCT-3´ |
| **Sigma factors** | *rpoD* | 5´-TGCGTATGCGTTTCGGTATC-3´ | 5´-GCGGGTAACGTCGAACTGTT-3´ |
|  | *rpoS* | 5´-CGCCGCCGGATGATC-3´ | 5´-CAGACCACGATTGCCATAACG-3´ |
| **Transcription factors** | *ihfA* | 5´-CGAAAACGGGCGAGGATA-3´ | 5´-CGACCCGGCTTTTTAACTTCT-3´ |
|  | *Crp* | 5´-TGTTTGAAGAGGGCCAGGAA-3´ | 5´-CCACTTCACAGGCGGTTTTC-3´ |
|  | *pdhR* | 5´-CATCGCCATCTGGCCTTTAT-3´ | 5´-CACGGCGGCTCTCTTCA-3´ |
|  | *fruR* | 5´-GGTTGGTGCCGATCAGGAT-3´ | 5´-TCTCGGCGGGAAACTTACG-3´ |
| **PPP** | *zwf* | 5´-GGCGCTGCGTTTTGCTAA-3´ | 5´-TTCTGCCACGGTAATCTCAACA-3´ |
| **Glucose transport** | *ptsG* | 5´-ACTGGCGTTGTGCTGTCCTT-3´ | 5´-GGTAAGCAGCCCACTGAGAGA-3´ |
| **Glycolysis** | *pykF* | 5´-CGAGTTCAACAATGACAACCGTAA-3´ | 5´-ATCAGCGGAGCATCCAGTTT-3´ |
|  | *pykA* | 5´-GCGCTGACCGAAAAAGACAA-3´ | 5´-CAGCCAGGTAATCTACGCCAAT-3´ |
|  | *aceE* | 5´-CCAAAGGCAAAGCGACAGTAAT-3´ | 5´-CCTGGTGCGCGATGTTTT-3´ |
| **Fermentation**  **Pathways** | *ldhA* | 5´-ACGGAAAAAACCGCTAAAACTG-3´ | 5´-ATATTTAACGCCGTGCTTTTTCA-3´ |
|  | *lldD* | 5´-GGGCTTGATGTCGTGCGTAT-3´ | 5´-AGAAAGCACGACCCAGCAGTA-3´ |
|  | *dld* | 5´-TCGTGGCTGGTGGATTATTTC-3´ | 5´-AACGCTTTGCTGCCTTCCT-3´ |
|  | *adhE* | 5´-GTGGTCCGGGCATGGTT-3´ | 5´-TTGCCCGCGCCTACAC-3´ |
|  | *pflB* | 5´-ACTGAATACCGTAAAACTCACAACCA-3´ | 5´-AGATTTACGGCAACGCAGGAT-3´ |
| **Acetate metabolism** | *acs* | 5´-AACACACCATTCCTGCCAACA-3´ | 5´-TGTTGATACATCGCCTCGTACTG-3´ |
|  | *actP* | 5´-ACCGGGTTTATGGGCTACTTCTA-3´ | 5´-CGGATTCGCACCAACCA-3´ |
|  | *ackA* | 5´-CGCGCAATGGACGTTTACT-3´ | 5´-TCCAGACGACCATCCATCAG-3´ |
|  | *poxB* | 5´-AGCGTGCTGGGCTTTGTG-3´ | 5´-TAGTTCGGTGCCGTCAGTCA-3´ |
| **TCA cycle** | *icdA* | 5´-AAGTTCACCGAAGGAGCGTTTA-3´ | 5´-GCCACCGTCGATCAGTTCA-3´ |
|  | *sucA* | 5´-GTCTCGCGTTGCCAAGATTTAT-3´ | 5´-GTGGCGTAAGCGAGGTTTTC-3´ |
|  | *sdhC* | 5´-CACATGATGATGGATTTTGGCTAT-3´ | 5´-GCACGACAGTAATAACAAAGGAGATTT-3´ |
|  | *mdh* | 5´-AGGCGCTTGCACTACTGTTAAAA-3´ | 5´-CGGGAGTCACTGGAGCGATA-3´ |
| **Glyoxylate shunt** | *aceA* | 5´-TGCACGGTGAGTCGAAAAAA-3´ | 5´-TAGACTGCTTCAATACCCGCTTT-3´ |
|  | *aceB* | 5´-TGGCGTGGTGAGGCAAT-3´ | 5’-GGAAGAAATAGAGCGCAAAATCA-3´ |
| **Gluconeogenesis and anaplerosis** | *maeB* | 5´-GAAGAGCTGGCGGAGATCAC-3´ | 5´-GCGGCTCAATACCAAAACGA-3´ |
|  | *sfcA* | 5´-TGCGGTTGGCAAAATGG-3´ | 5´-AGGGCTTCGGCAGAGGTT-3´ |
|  | *pck* | 5´-CGTCTTTCCGTCCGTTTCAT-3´ | 5´-GCTCGGGCGAATAAACATGT-3´ |
|  | *ppc* | 5´-GGAAGAGAACCTCGGCTACAAA-3´ | 5´-CCGCCCATCCACGAAGT-3´ |
|  | *pps* | 5´-CCTCCCTGGGTGAAATGATTACTA-3´ | 5´-GGCGGTTGTGGCGAAA-3´ |
